# PM_10_ Plume dispersion data of the Zouk power plant in Lebanon

**DOI:** 10.1016/j.dib.2018.09.047

**Published:** 2018-09-19

**Authors:** Samer Salloum, Julie Nassar, Rima Baalbaki, Alan L. Shihadeh, Najat A. Saliba, Issam Lakkis

**Affiliations:** a Department of Mechanical Engineering, Faculty of Engineering and Architecture, American University of Beirut, Beirut 1107 2020, Lebanon; b Department of Chemistry, Faculty of Arts and Sciences, American University of Beirut, Beirut 1107 2020, Lebanon

**Keywords:** Ambient air pollution, PM10, Zouk Mikael power plant, TAPM

## Abstract

Ambient air pollution is a major risk to the human health and to the environment. The data presented quantifies the (PM_10_) contribution of the Zouk Mikael power plant to the ambient air pollution in Lebanon for the year 2014. The data is the outcome of a computer simulation using The Air Pollution Model (TAPM), taking into account the emission source data, the spatio-temporal meteorological conditions, the terrain height, and the land cover characteristics. The data set presents the annual, seasonal and monthly averages of the spatial distribution of the ground-level particulate (PM_10_) concentrations in the ambient air. The data set also includes spatial distribution of the maximum concentrations, which revealed two zones of elevated concentrations. Monthly averages and maximum concentrations in these two zones are also reported. Analysis of the data can provide information on the health risk the residents in the affected areas are subjected to. The data can also provide insight on the impact of the meteorological conditions (temperature and velocity) and the topography on pollutant dispersion in regions bounded by the sea and by a mountain range.

## Specifications table

**Table:** 

*Subject area*	*Modeling and simulation*
*More specific subject area*	*Air Pollution Modeling*
*Type of data*	*Figures and barcharts*
*How data was acquired*	*Computer simulation using The Air Pollution Model (TAPM)*
*Data format*	*Analyzed*
*Experimental factors*	• *The ratio of Total Suspended Particles to PM10 is assumed to be 0.5* [2]*.* • *An ideal scenario of regular maintenance is assumed.* • *Although the complex operating schedule of the power plant is not taken into account, the average emitted mass flow rate is conserved.*
*Experimental features*	• *The Air Pollution Model takes as its input (1) the terrain height data, land cover characterization data, soil texture types, mean sea surface temperatures, (2) six-hourly synoptic scale 4D conditions for the year 2014 and (3) the emission sources locations, dimensions, chemical composition, flowrate, and thermophysical conditions.*
*Data source location*	*Zouk Mikael, Lebanon (33.9703°N, 35.6206°E)*
*Data accessibility*	*Data is with this article*
*Related research article*	*[1] Rima Baalbaki, Julie Nassar, Samer Salloum, Alan L. Shihadeh, Issam Lakkis, Najat A. Saliba,“Comparison of atmospheric polycyclic aromatic hydrocarbon levels in three urban areas in Lebanon”, Atmospheric Environment, Volume 179, 2018, Pages 260–267, ISSN 1352–2310,*https://doi.org/10.1016/j.atmosenv.2018.02.028.

## Value of the data

● The data could be used to assess the contribution of the power plant to the pollution in the nearby region.
● The data can be used to identify zones of elevated PM_10_ concentrations.
● The data can be used to decide on distribution and locations of pollution monitoring sensors.
● The data can be used in conjunction with long term measurements to improve pollution forecast for both the short term as well as the long term.
● The data can be used to carry out geographically based health risk assessment.
● The data can be used to decide on sample size and distribution for correlating pollution exposure with incidences of diseases.
● The data can be used by environmental scientists to gain insight into seasonal variations of the pollution dispersion in similar settings.
● The data can be used by researchers in environmental policy to propose changes in exisiting conditions and/or propose changes in the policies for the future.

## 1 Data

TAPM outputs show the power plant stack emission trajectories for a whole year. An animation can be found through the following link https://www.youtube.com/watch?v=aml0oELAdBA.

**Fig. 1:**
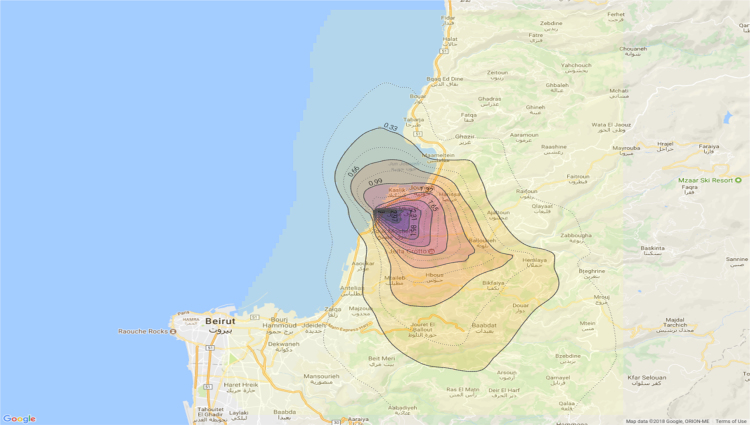
Average annual PM_10_ concentration in μg/m^3^ for the year 2014.

**Fig. 2:**
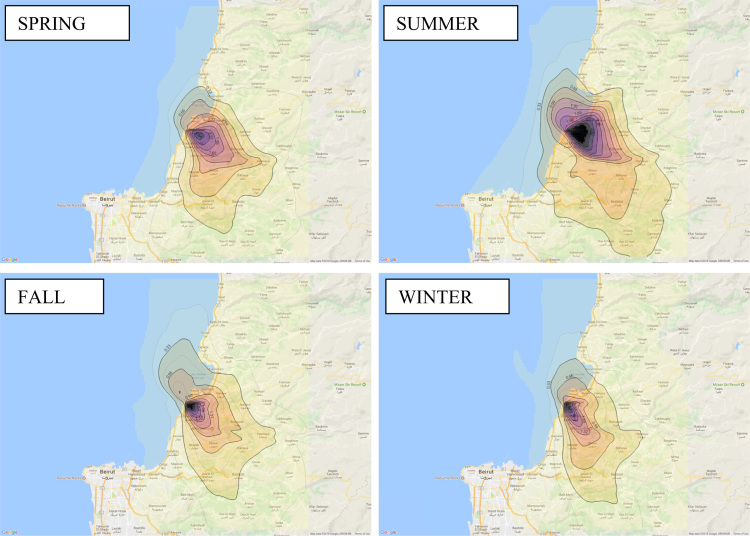
Average seasonal PM_10_ concentration in μg/m^3^ for the year 2014.

**Fig. 3:**
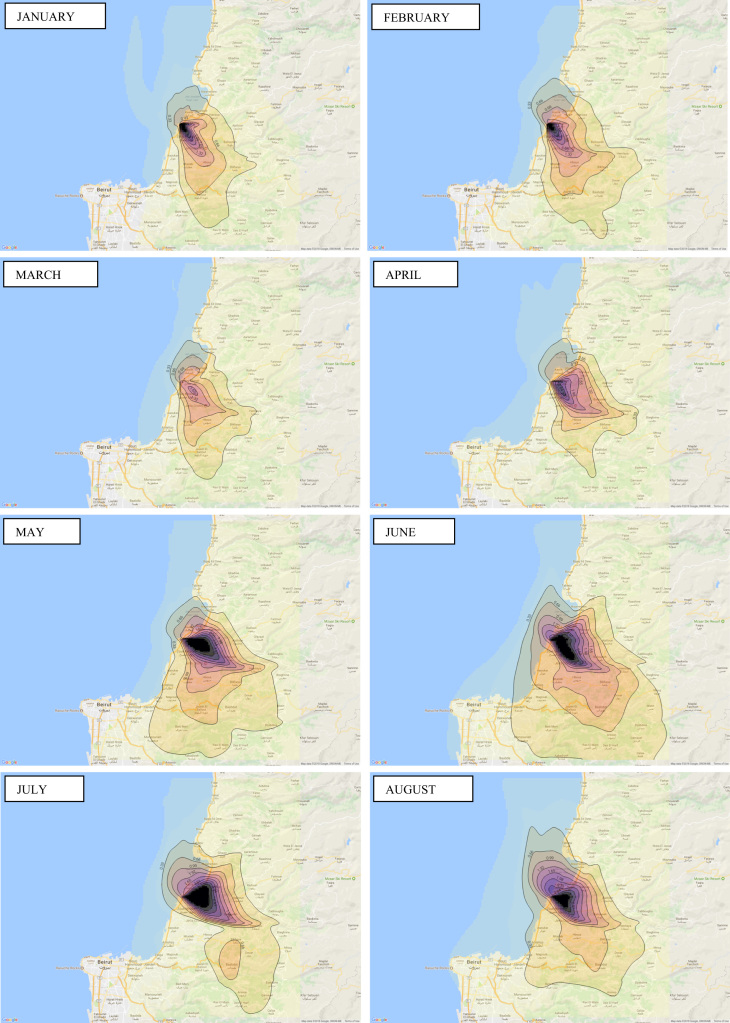

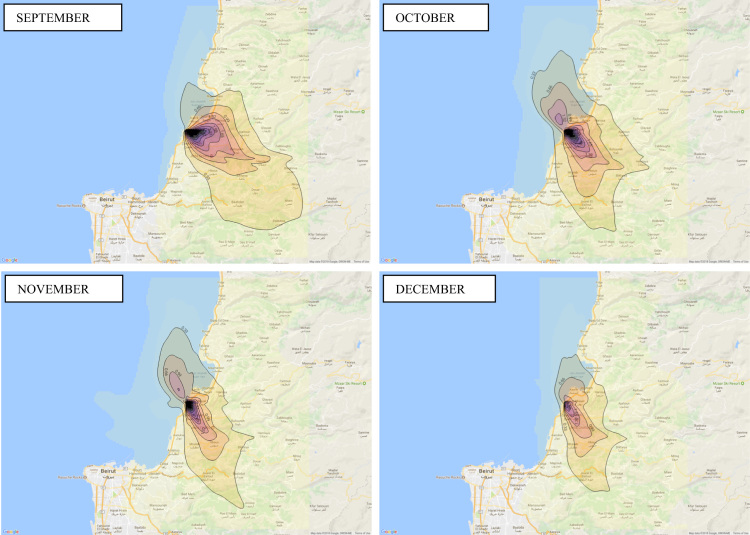
Average monthly PM_10_ concentration in μg/m^3^ for the year 2014.

**Fig. 4:**
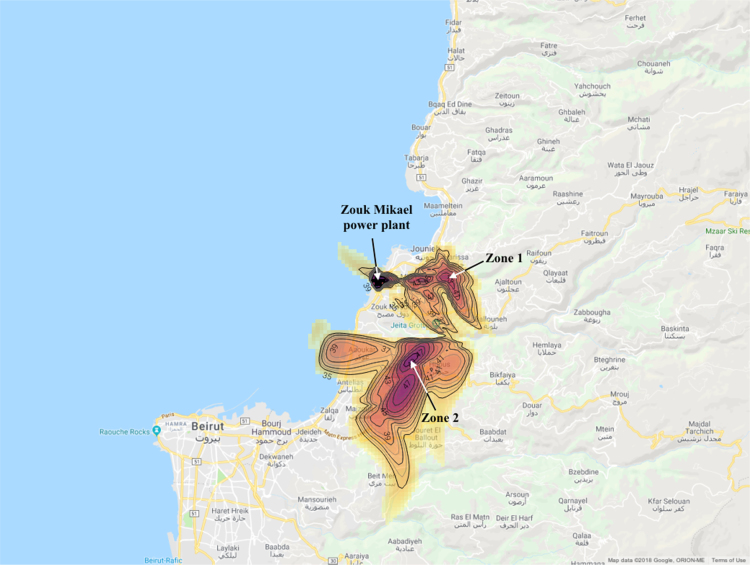
Maximum annual PM_10_ concentration in μg/m^3^ for the year 2014 showing two zones of elevated PM_10_ concentrations.

**Fig. 5:**
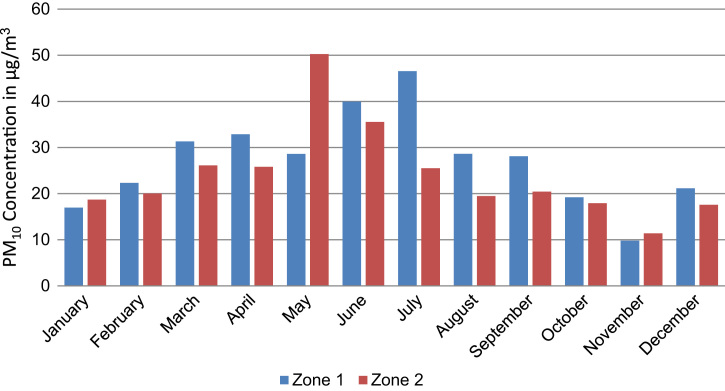
Maximum PM_10_ concentration in μg/m^3^ at Zone 1 and Zone 2 for every month in 2014.

**Fig. 6:**
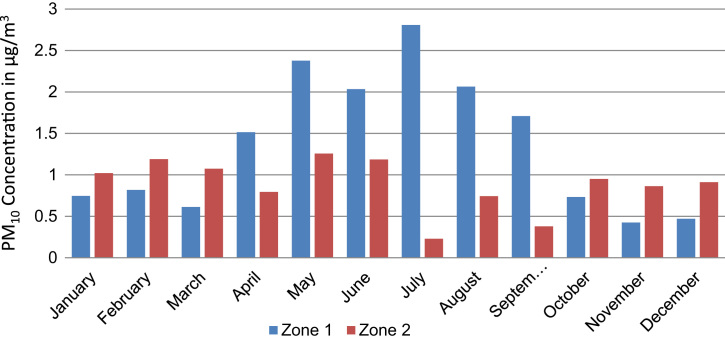
Average PM_10_ concentration in μg/m^3^ at Zone 1 and Zone 2 for every month in 2014.

Annual average PM_10_ concentration map at ground level is shown in Fig. 1. Seasonal average PM_10_ concentration mapsat ground level are shown in Fig. 2. Monthly average PM_10_ concentration maps at ground level are shown in Fig. 3. Fig. 4 shows the map of the maximum annual PM_10_ concentration, where two distinct zones of elevated maximum concentrations are identified. Fig. 5 presents a plot of monthly variation of the maximum PM_10_ concentration in Zones 1 and 2. Fig. 6 presents a plot of monthly variation of the average PM_10_ concentration in Zones 1 and 2.

## 2 Experimental design, materials, and methods

PM_10_ emission rate are calculated from data provided by EDL Engineers as described in the Related research article [1]. Geometric characteristics of the plant turbines and stacks as well as the plant running schedule are also detailed in the Related research article [1]. A summary is presented here:

Emissions source:

○ four stacks, each stack is 9 m in diameter and 145 m high.
○ On average, 2.5 out of 4 stream turbines operate continuously.
○ PM_10_ emissions of 10.2 g/s through the stacks at a temperature of 700 K.

The Air Pollution Model [3], [4] (TAPM) is a computational tool that predicts pollutant transport, taking into account atmospheric chemical reactions, and species advection by weather flow field patterns [4]. TAPM numerically solves an approximation of the Navier Stokes (N–S) equations to predict the wind velocity field over a prescribed period of time and in a selected region. The wind velocity field is then used as an input to solve the species transport equation in order to obtain the hourly concentration profile for the pollutants. The model, which employs nested grids, starts solving the governing equations on the outermost grid, where the boundary and initial conditions are obtained from global synoptic analyses. It then passes the large-scale information as boundary conditions to the next (finer) grid. Radiative fluxes, both at the surface and at upper levels, are also included [4]. The soil temperature and moisture content are calculated following the method presented in [5] using some empirically derived constants or functions of soil moisture content and soil texture type.
